# Advanced mapping of inorganic treatments on porous carbonate stones by combined synchrotron radiation high lateral μXRPD and μXRF

**DOI:** 10.1038/s41598-024-58718-z

**Published:** 2024-04-20

**Authors:** G. Massinelli, N. Marinoni, C. Colombo, G. D. Gatta, M. Realini, M. Burghammer, E. Possenti

**Affiliations:** 1 Dipartimento di Scienze della Terra “Ardito Desio”, Università degli Studi di Milano, Via Botticelli 23, 20133 Milan, Italy; 2 Istituto di Scienze del Patrimonio Culturale (ISPC), Consiglio Nazionale delle Ricerche (CNR), Via R. Cozzi 53, 20125 Milano, Italy; 3 European Synchtron Radiation Facility, 71 Avenue des Martyrs, 38000 Grenoble, France

**Keywords:** Materials science, Characterization and analytical techniques

## Abstract

Understanding the effects of consolidating inorganic mineral treatments on carbonate stones of cultural heritage, and on the nature and distribution of newly formed products within the matrix, poses a significant challenge in Heritage Science and Conservation Science. Existing analytical methods often fail to deliver spatial and compositional insights into the newly formed crystalline phases with the appropriate high lateral resolution. In this study, we explore the capabilities and limitations of synchrotron radiation (SR) micro-X-ray powder diffraction (μXRPD) mapping combined with micro-X-ray fluorescence (μXRF) to give insight into compounds formed following the application of ammonium oxalate (AmOx) and diammonium phosphate-based (DAP) solutions on porous carbonate stone. Ultimately, the integration of μXRPD mapping and μXRF analysis proved itself a powerful asset in providing precise qualitative and quantitative data on the newly formed phases, in the case of both calcium oxalates (CaOxs) and calcium phosphates (CaPs), and their complex stratigraphic distribution, thus opening a new route for applications to a more comprehensive study of inorganic treatments applied to carbonate substrates.

## Introduction

The consolidation of carbonate stones poses a significant challenge in preserving Cultural Heritage (CH) buildings and artifacts due to material decohesion caused by decay^1^. Since the mid-1990s, various inorganic treatments have been developed to restore microstructural cohesion within damaged stones matrixes^2–4^. In such context, ammonium oxalate (AmOx) and di-ammonium phosphate (DAP) solutions have gained prominence for carbonate stones in the past two decades^5–7^. These water-soluble salts react with the carbonate matrix of the stone substrate to produce micrometric reaction products (ranging from 0.1 to 4 µm in crystal size)^8^. Specifically, AmOx primarily produces Ca-oxalate (CaOxs) while DAP's reaction should ideally yield calcium phosphate phases (CaPs). Oxalate phases exhibit limited penetration into the stone (~ 1 mm) whereas DAP-based treatments offer greater consolidating capabilities in terms of treatment penetration (2–8 mm, depending on the stone substrate)^9,10^.

However, inorganic mineral treatments exhibit significant variability in terms of the newly formed phases within the treated stone materials and their effect on the stone microstructure. Factors such as their crystal-chemical composition, crystallinity, penetration, and distribution are influenced by a series of interrelated variables playing during the solution reaction with the calcite matrix (i.e., solution molarity, treatment methodology, etc.)^10,12^*.* Consequently, studying the outcome of the reaction process, specifically the new “reaction products-calcite matrix” system, presents a significant challenge. Despite this complexity, understanding the modifications induced by inorganic treatments is crucial, given that the physical, chemical, and microstructural properties of the new system significantly impact the macroscopic interaction of the building stone with the agents of decay. The complexity of studying these transformations becomes particularly pronounced in the case of reaction products that share a similar elemental composition to those of the carbonate stone substrate, like in the case of CaOxs formed by AmOx solutions, or in the case of complex reaction kinetics in the presence of carbonate ions that result in not stoichiometric reaction. Examples of this last scenario are CaPs phases formed with DAP solutions, which ideally should yield crystalline hydroxyapatite (HAP, Ca_10_(PO_4_)_6_(OH)_2_), however, the result is complex mixtures of crystalline and poorly crystalline phosphate phases^11^*.*

In accordance, a range of analytical techniques has been employed tailored to characterize carbonate stone treated with inorganic mineral treatments and gain insights into the composition and penetration depth of the newly formed phases to assert their performance. Conventional methods such as SEM–EDX, XRD, and FT-IR have been widely used but lack the specificity and lateral resolution required for characterizing phases with different degrees of crystallinity, in addition to being invasive (as many of their experimental setups require a sample) or, in some cases destructive (i.e., with a complex sample preparation)^13–15^. High lateral resolution micro-spectroscopy techniques, like μ-ATR-FTIR and μ-Raman mapping or spectroscopic imaging, have proven valuable for probing the spatial distribution of consolidants, providing complementary structural and compositional insights on the newly formed phases^11,16–18^. However, these techniques may face challenges in distinguishing the newly formed phases within the microstructure due to overlapping vibrational bands. More recently, synchrotron radiation (SR) facilities have experienced a remarkable rise in the study of Cultural Heritage materials^19–22^. Advanced radiation imaging techniques, such as SR-based X-ray micro-computed tomography (μ-CT), have been employed to non-invasively determine the effects of the new product crystallization on the 3D stone’s microstructure at a maximum voxel size of 1 μm^3^. However, no chemical information is provided^23–27^. Moreover, micro-X-ray diffraction computed tomography (XRDCT) measurements allow structural characterization and 3D localization of the reaction products but may lack point-specific information at good spatial resolution^28,29^. Lastly, more recently, SR-based spectroscopy techniques such as Ca K-edge 2D X-ray absorption near-edge structure (XANES), with μ-XRF mapping, have been successfully employed to spatially distinguish among amorphous calcium carbonate with a micrometric lateral resolution^30^.

Despite the advancements in analytical techniques, it is still challenging to achieve comprehensive spatially resolved insights into the “reaction products-calcite matrix” system. Synchrotron radiation (SR) micro-X-ray powder diffraction (μXRPD) mapping stands out as a powerful asset, as it can provide spatially resolved information about the crystallographic properties of the reaction products at a lateral resolution down to the submicrometric scales. This technique, when complemented with the elemental information of micro-X-ray fluorescence (μXRF) mapping, becomes particularly well-suited for the micro-analysis of highly heterogeneous systems, especially when the new phases are present in minor fractions if compared to the substrate’s minerals or are poorly crystalline. These techniques, although well-known and already used in many fields over the years^31–35^, in CH materials are mainly used in the examination of oil paintings^22–39^ and wall paintings^40,41^, ceramics^42,44^, paper^45^, and metals^46,47^, but, to the best of our knowledge, have not been employed to study the reaction products of inorganic consolidation solutions on porous carbonate stones in CH.

In this paper, by building on what others have done in this field, we’re also bringing something new to the table by addressing the challenges of dealing with more complicated systems. We explore the capabilities and limitations of this methodology approach combining SR-μXRPD and μXRF mapping, in collecting comprehensive qualitative and quantitative information about the crystal-chemical compositions and spatial distribution of the newly formed phases (CaPs and CaOxs), resulting from the reaction of AmOx and DAP solutions on porous carbonate stones, to test its applicability and contribution in the ongoing knowledge related to stone conservation in Cultural Heritage.

## Materials and methods

### Sample selection and preparation

Noto Yellowish Limestone, our primary substrate, is a building stone used for Baroque monuments in the Val di Noto (south-eastern Sicily) with a predominant composition of calcite, with minor clay minerals, quartz, and iron hydroxides. This stone shows a pore size range from 1 to 10 μm and from 1 to 0.1 μm and an open porosity ranging between 25 and 35%^24,29^.

A tailored preparation method was devised to preserve crystalline phases and microstructural information. A thin section, of approximately 30 μm thickness, was prepared traversal (from the treated surface to the bulk) from each 5 × 5 × 2 cm^3^ treated limestone specimen. Embedding the sample in resin and adding a piece of polycarbonate to the section were essential steps to stabilize the thin section, during the follow-up cutting procedure. The thin sections were resized to a final size of 2 × 4 mm, to optimize the mounting space on the sample holders. Such preparation is compatible with the ID13 set-up allowing for optimal transmission of X-rays and a controlled and uniform probed voxel across the two-dimensional surface.

To obtain a more objective assessment of the capabilities and limitations of the techniques employed, we subjected the limestone samples to various treatment protocols. This systematic approach allows us to assess the performance of our methodologies under different conditions. Two protocols encompassed the application of just one solution (either AmOx or DAP). Another one adopted the application of a sequential treatment (DAP followed by AmOx solution). The diverse treatments aim to replicate real-world situations encountered in the conservation of CH materials. Concentrations for DAP and AmOx as well as application methodology and condition were determined based on precedent experiments and established practices from conservation worksites.

### Combined micro X‑ray fluorescence (µXRF) and micro X‑ray powder diffraction (µXRPD)

**Figure 1:**
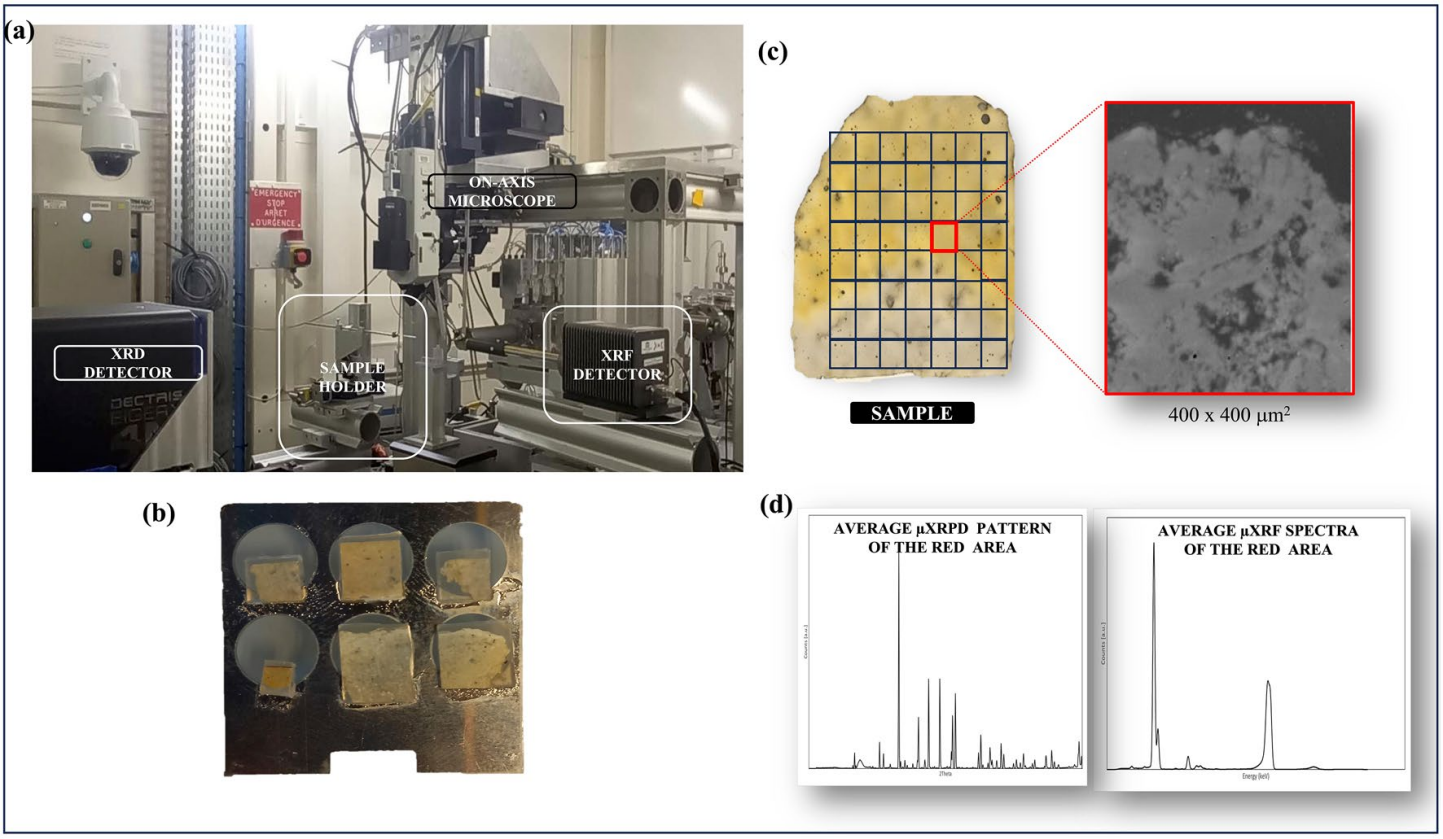
(**a**) SR-µXRPD scanning and µXRF set-up at ID13 beamline, ESRF, (**b**) some of the analyzed thin section (2 × 4 mm) mounted on the 4 mm diameter 12-hole holder, and (**c**) Schematic illustration of μXRPD mapping and μXRF experiment on Noto limestone thin section. (**d**) µXRD patterns and µXRF spectra were acquired at each pixel of an area of 400 μm (horizontal) by 400 μm (vertical) defined over the sample surface.

The combined Micro X‑ray fluorescence (µXRF) and Micro X‑ray Powder diffraction (µXRPD) investigations were conducted at the ID13 beamline at the European Synchrotron Radiation Facility (ESRF) in Grenoble (Fig. 1a). The samples were mounted together on a 4 mm diameter 12-hole holder, as shown in Fig. 1b, then mounted vertically, perpendicular to the X-ray beam. The μXRPD branch performed crystalline phase mapping using a 2.5 × 2.5 μm^2^ beam with an energy of 13 keV. Throughout the thin section, three regions of interest (ROIs) were systematically collected, commencing from the sample surface (as illustrated in Fig. 1c). Each ROI corresponds to a map size of 400 μm (horizontal) by 400 μm (vertical) covering an area of the sample of approximately 1.2 mm. This specific ROI size was chosen to strike a balance between achieving sufficient spatial detail and maintaining manageable data acquisition times. Considering the characteristics of the limestone samples, including potential variations in crystalline phases or microstructural features, the 400 μm by 400 μm ROI was deemed apt for capturing relevant information without the pitfalls of oversampling or undersampling. Two-dimensional (2D) diffraction patterns were acquired in transmission mode at every pixel of 2D maps using the graphical user interface (GUI) Daiquiri. These X-ray diffraction patterns were subsequently transformed into one-dimensional (1D) diffractograms through azimuthal integration, facilitated by Jupyter Notebooks based on the PyFAI software package^46^. Simultaneously to each diffraction map, μXRF spectra were obtained for the same map (Fig. 1d), using a Vortex EM detector mounted orthogonally to the beam.

### Data processing

Data processing was an essential step in our study as the “reaction products–stone matrix” system exhibited Ca-baring elemental composition but with diverse crystalline phases in different locations and degrees of crystallinity. To investigate this complexity, we relied on the PyMca 5.9.2 ROI imaging software (link https://sourceforge.net/projects/pymca/)^48^, which enables simultaneous visualization of µXRPD and µXRF imaging data. This parallel processing workflow maximized the synergy of the two techniques, allowing us to not only visualize elemental composition but also directly identify crystalline phases and their location.

**Figure 2:**
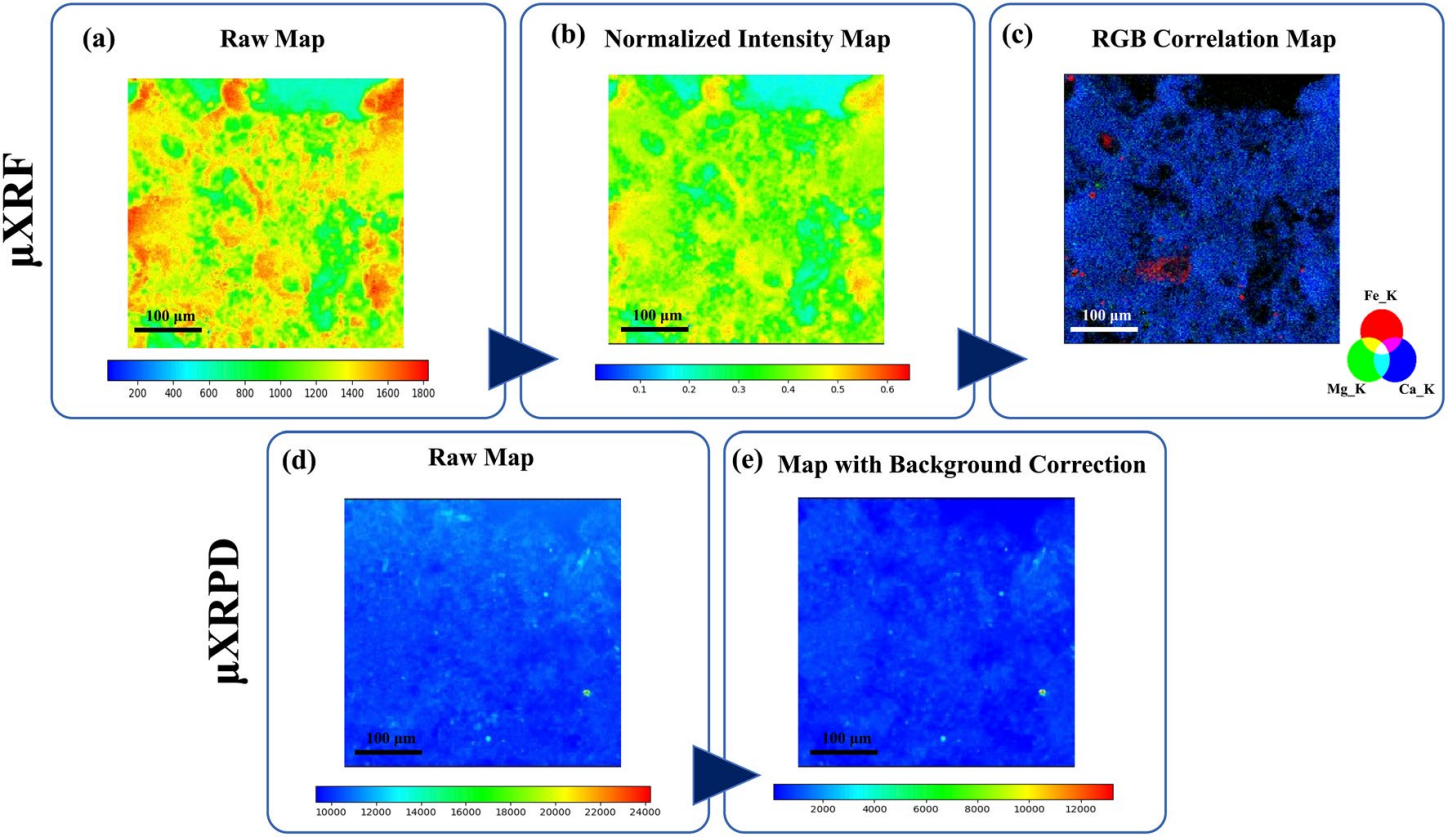
Data processing on the PyMca ROI imaging software of one of the samples. (**a**) Original μXRF map of an ROI; (**b**) I/I_0_ normalization of the μXRF map; (**c**) RGB correlation of the elemental distribution maps of calcium, iron, and magnesium; (**d**) Original μXRPD map of the same ROI and (**e**) μXRPD map after the 1D background correction.

The initial data processing step involved I/I_0_ normalization of the intensity map pixels to rectify variations induced by the incoming X-ray beam. Figure 2a shows an example of the false color appearance of the µXRF map before normalization, emphasizing the importance of this step to produce an accurate representation of elemental intensity (in Fig. 2b the elemental map after the normalization is shown).

Afterward, the normalized μXRF map underwent a fitting process for specific elements composing our “reaction products-calcite matrix” system. The fitting process generated distribution maps for these elements, enabling effective comparisons of their presence and distribution. An illustrative example Fig. 2c displays the elemental distribution map for calcium in one of the samples.

Regarding the μXRPD imaging, the software displayed a correlated µXRPD pattern representing the averaged profile of all acquired patterns across the 2D map. Raw data preprocessing involved 1D background correction, addressing the contributions of the amorphous polycarbonate layer in the sample preparation, which can make it more challenging to identify and analyze the diffraction peaks associated with the limestone system. This improvement in peak-to-background ratio facilitates more reliable peak identification, especially for weak or overlapping peaks, like in the case of the new crystalline phases. The exclusion of low-angle regions was done to not result in the loss of important information associated with low angles, like some amorphous or poorly crystalline phases of CaPs and CaOxs. In Fig. 2d,e, the effect on μXRPD maps of the background correction process is shown.

In the post-preprocessing, the identification of crystalline phases within µXRPD patterns, exported from PyMca, employed the X’Pert Highscore Plus software 5.2 (link https://www.malvernpanalytical.com/en/support/product-support/software/highscore-software-update). The analysis complemented the data processing using PyMca. Leveraging the strengths of PyMca, we conducted tailored data post-processing to address the diverse analytical inquiries arising in the study of the effects of inorganic treatments on carbonate stones. The subsequent post-processing methodologies will be described in the upcoming sections.

PyMca’s “RGB Correlator” tool was mainly used as it visually highlights distribution patterns of elements alongside crystalline phases, creating µXRPD and µXRF correlation maps. This capability proved indispensable in unraveling relationships between element distribution and identifying correlations or anticorrelations in calcium-based crystalline phases within a predominantly calcite matrix.

Finally, the quantification of XRD data involved image analysis using Fiji, Open-Source software ImageJ (OS version, link https://imagej.net/ij/), applied to diffraction maps corresponding to each identified phase. Utilizing the threshold tool, the software extracted the areal percentage of each phase within the specified region of interest. This process provided a quantitative distribution of each phase. To ensure the robustness of our findings, average percentages and their standard deviations were calculated for each treatment. Also, to mitigate subjectivity associated with threshold tools, small squares were inserted inside the areas designated for segmentation, and all the data were plotted and described by a function. The data is well-fitted with a Gaussian function. The limits of this Gaussian function were employed during the segmentation. Furthermore, all images processed with this method were acquired under the same experimental conditions and saved with consistent dimensions and resolutions to maintain constant image quality.

## Results and discussion

### Qualitative phase characterization of the “reaction products-calcite matrix” system

Initially, we explored the combined µXRPD and µXRF mapping capabilities to characterize the different reaction products (CaOxs and CaPs) within the newly formed system for all treatment methodologies (single treatment and sequential treatments).

**Figure 3:**
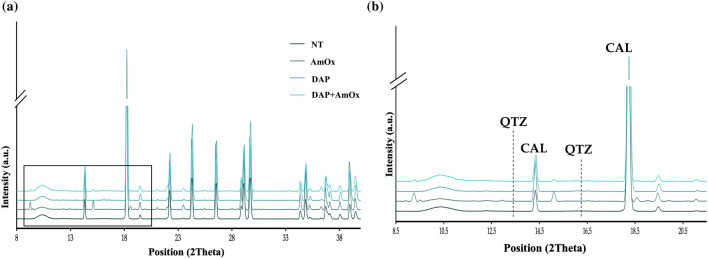
(**a**) Average μXRPD patterns extracted from representative surface maps of an untreated Noto limestone sample (NT) and three samples treated by AmOx, DAP, and DAP + AmOx; (**b**) A zoom (position ~ 8–20 2Theta) to highlight the marker peaks of calcite (CAL) and quartz (QTZ).

To begin, we analyzed the average µXRPD patterns for all Noto Limestone samples, both untreated and treated. The average diffraction patterns of the untreated limestone samples show predominantly the peaks associated with calcite, with occasional quartz. This trend remains consistent across treated samples’ diffraction patterns, as illustrated in Fig. 3. In these patterns, the main peaks of the reaction products were weak. This result aligns with prior literature and reflects how the reaction products of both solutions are in a low wt% fraction if compared to the abundant calcite matrix^17^.

**Figure 4:**
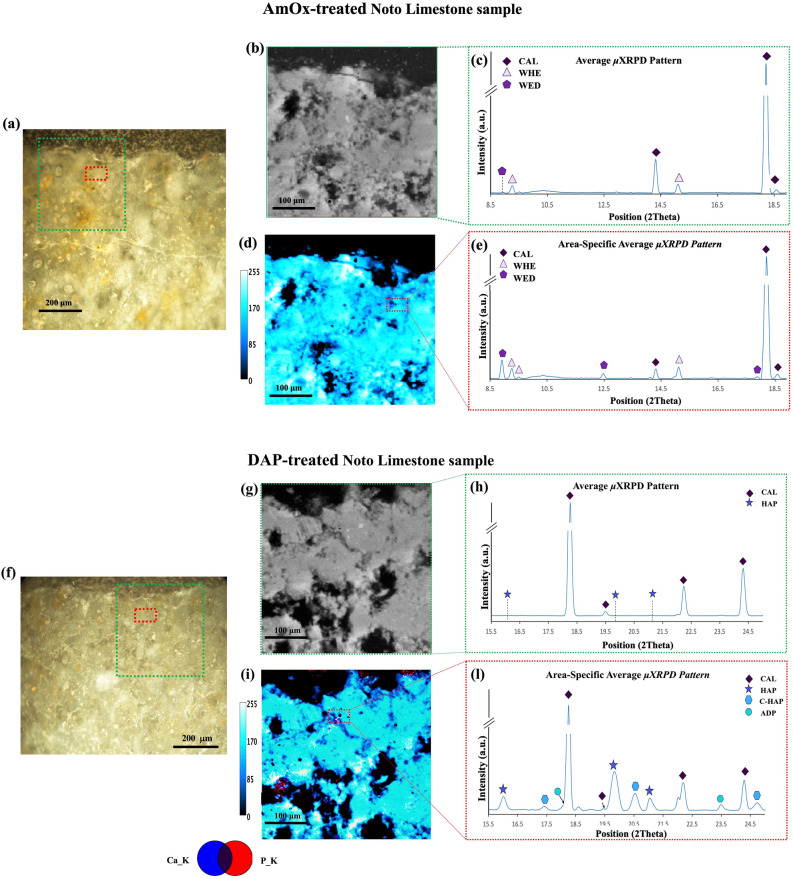
Comparison between single treated samples. For the AmOx treated: (**a**) Optical image of the cross-section. Selection of the investigated ROI (green) and the investigated pixels (red); (**b**) Representative average surface μXRF map of the ROI; (**c**) Map average μXRPD pattern extracted from the ROI; (**d**) Elemental map of calcium showing variation in intensity; (**e**) Area-specific average μXRPD pattern for the red area exhibiting low Ca intensity. A zoom of the diffraction patterns (position ~ 8–20 2Theta) is shown to highlight the marker peaks of whewellite (WHE) and weddellite (WED). For the DAP treated: (**f**) Optical image of the cross-section. Selection of the investigated ROI (green) and the investigated pixels (red); (**g**) Representative surface μXRF map of the ROI; (**h**) Map average μXRPD pattern extracted from the ROI (**i**) RGB correlation map of the elemental distribution of calcium (blue) and phosphorous (red); (**l**) Area-specific average μXRPD pattern for the red area exhibiting correlation Ca/P. The patterns are presented in a zoom (position ~ 16–25 2Theta) to highlight the marker peaks hydroxyapatite (HAP), Ammonium Hydrogen Phosphate (ADP), and carbonate- substituted HAP (C-HAP).

Focusing specifically on the reaction products from the AmOx solution (Fig. 4a and b), the CaOxs, low-intensity peaks appear in some surface-proximate diffraction patterns (as shown in Fig. 4c), where the highest crystallization activity for these phases usually occurs, suggesting the presence of substances, but not a conclusively identification. The challenge in their identification arises due to the nano-crystalline nature of the main CaOx phases—whewellite (CaOx monohydrate) and weddellite (CaOx dihydrate)—together with their low fractions if compared to the calcite matrix^44^. Even with our high-resolution XRPD patterns, discerning these CaOxs in all patterns remains difficult.

The sole examination of μXRF imaging does not facilitate their identification either, as CaOxs share a similar elemental composition to the calcite matrix (Ca, C, O). However, in the case of the AmOx-treated samples, the high-resolution μXRF Ca maps have shown, as seen in Fig. 4d, low-intensity (~ 70–100 in the scale bar) and high-intensity areas (corresponding to ~ 170–250 in the scale bar). An unusually big gap in the intensity of Ca through the elemental map was here registered. These intensity variations throughout the Ca maps could, therefore, be correlated with different calcium contents in crystalline phases within our “reaction products-calcite matrix” system. Given that CaOxs contain less calcium (around 38% CaO) than calcite (approximately 55% CaO)^52^, these intensity fluctuations in the elemental map could suggest a different distribution of oxalate and calcite phases within the stone system, corresponding respectively to the low and high-intensity area.

Combining the μXRF maps with μXRPD data, we could generate *area-specific* average diffraction patterns by selecting pixels from areas exhibiting these calcium intensity variations. These patterns reveal peaks attributed to CaOxs with good resolution, as shown in Fig. 4e. The *area-specific* diffraction pattern exhibited a high signal-to-noise ratio and a consistently flat baseline, which helps us confirm not only the crystallization but also facilitates the unambiguous identification of whewellite and weddellite.

The situation becomes even more complex in the case of DAP-treatment samples (Fig. 4f and g). Like CaOxs, CaPs are nanometric-sized products and low in concentration^49^. Additionally, the complex reaction of DAP solutions with calcite does not yield only hydroxyapatite (HAP) but results in a mixture of by-products with varying Ca/P ratios in concentration distributed within the matrix based on reaction conditions^50^. These by-product phases share similarities in elemental composition and crystalline structure^51^, making their phase identification even more complex. Consequently, map average diffraction patterns of DAP-treated samples lack identifiable peaks even in our diffraction patterns, as shown in Fig. 4h, which hinders the confirmation of the presence of CaPs.

Moving on to μXRF imaging, a situation like the AmOx-treated sample was observed in the elemental maps of calcium distribution in samples treated with DAP. This is coupled with localized high phosphorus intensities (Fig. 4i). Given the known high Ca/P ratio in HAP (around 1.76), correlating calcium intensity variations with areas of high phosphorus concentration implies the potential CaP presence. Confirming this involves associating the μXRF imaging with the μXRPD data, generating *area-specific* diffraction patterns for pixels showing Ca/P elemental correlation. By generating specific diffraction patterns for these correlated areas, the identification of CaPs was achievable. The resulting diffraction patterns showed well-resolved HAP peaks that supported its visualization and identification. These patterns exhibit also good resolution at the low angular range where by-products of the reaction reside. The result is that this combined technique aids in identifying traditionally difficult-to-characterize^20^ by-products such as octacalcium phosphate (OCP) and other CaP phases associated with HAP crystallization, such as Ammonium Dihydrogen Phosphate ADP, and carbonate-substituted HAP (C-HAP), as shown in Fig. 4l, providing crucial information about the composition of the DAP “reaction products-calcite matrix” system.

**Figure 5:**
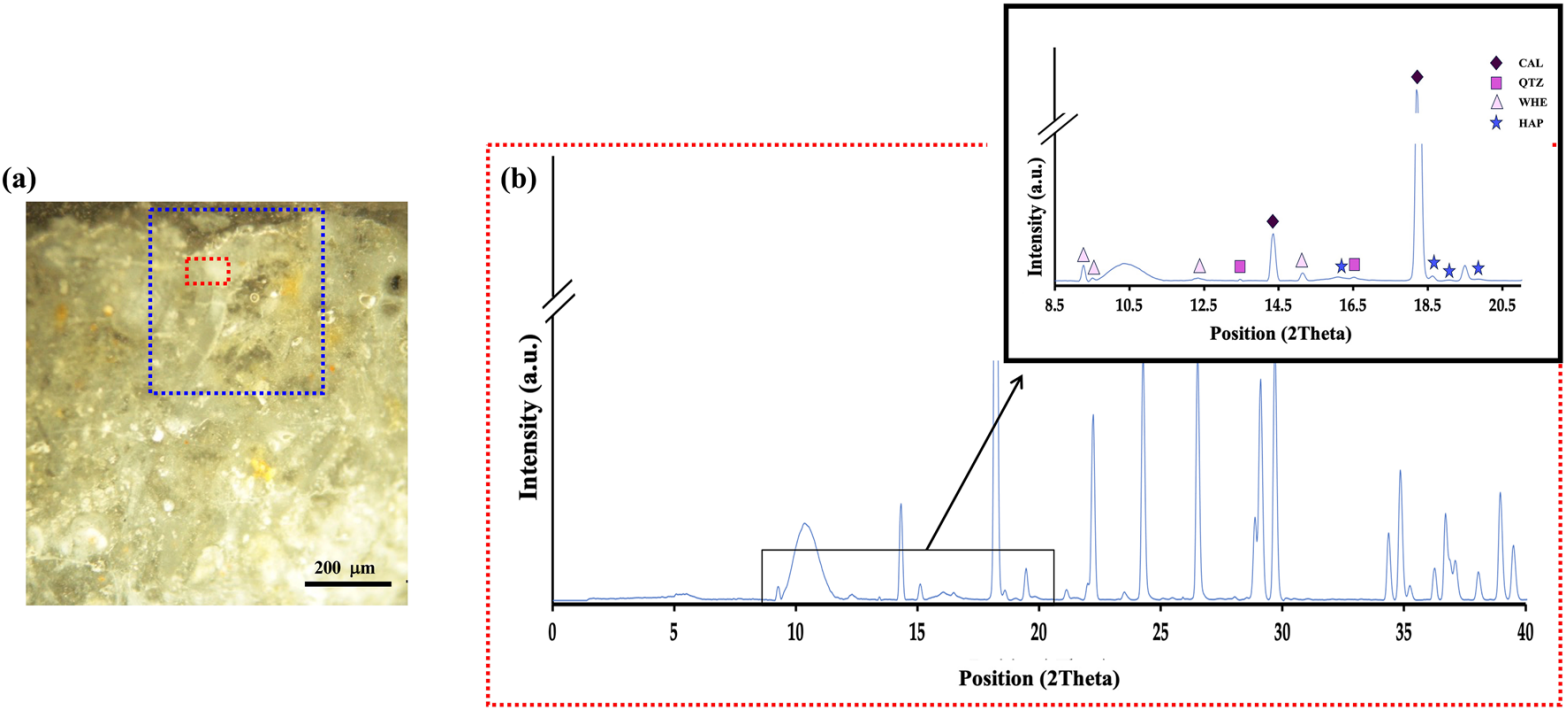
DAP + AmOx treated Noto Limestone sample (**a**) Optical image of the cross-section. Selection of the investigated ROI (blue) and the investigated pixels (red); (**b**) Area-specific average μXRPD pattern for the red area and a zoom (position ~ 8–21 2Theta) to highlight the marker peaks of hydroxyapatite (HAP), whewellite (WHE), and weddellite (WED).

The combination of techniques proves effective for qualitative phase analysis of both CaOxs (whewellite and weddellite) and CaP (HAP and by-products), even in the more complex scenario of sequential treatment samples (DAP + AmOx). Despite the additional challenges posed here because of the simultaneous presence of CaOxs and CaPs, this approach facilitated the visualization and characterization of the newly crystallized phases (oxalate and phosphate) within the complex systems generated by these treatments, as shown in Fig. 5.

In the end, focusing solely on the average μXRPD pattern, the identification of reaction products was limited mainly because of their low concentration compared to the dominant calcite matrix. In contrast, examining μXRF data alone resulted challenging in samples lacking elemental markers for identifying CaOxs and could only hint at the crystallization of CaPs when the markers were present. Integrating both techniques enabled the production of *area-specific* diffraction patterns associated with those zones exhibiting calcium intensity variations or high Ca/P correlation, allowing for the identification of specific crystalline phases characteristic of the newly formed “reaction products-calcite matrix” system.

### Phases’ penetration depth and spatial distribution of the reaction products

The combination of techniques used in this study yields an invaluable advantage in generating comprehensive μXRPD maps. *Phase-specific* distribution maps were generated by isolating distinct peaks associated with the identified crystalline phases in the diffraction patterns, providing detailed insight into the spatial distribution of each phase. SR-μXRPD mapping allows micrometric individual mapping of each reaction product distribution within the “reaction products-calcite matrix” systems, offering good spatial resolution. Such data not only visualizes the penetration depth of the crystallization but also localizes where the chemical reactions take place.

Our analysis consistently reveals *phase-specific* color maps accurately depicting the areal distribution of CaOxs and CaPs crystalline phases across all examined samples, without artifacts.

**Figure 6:**
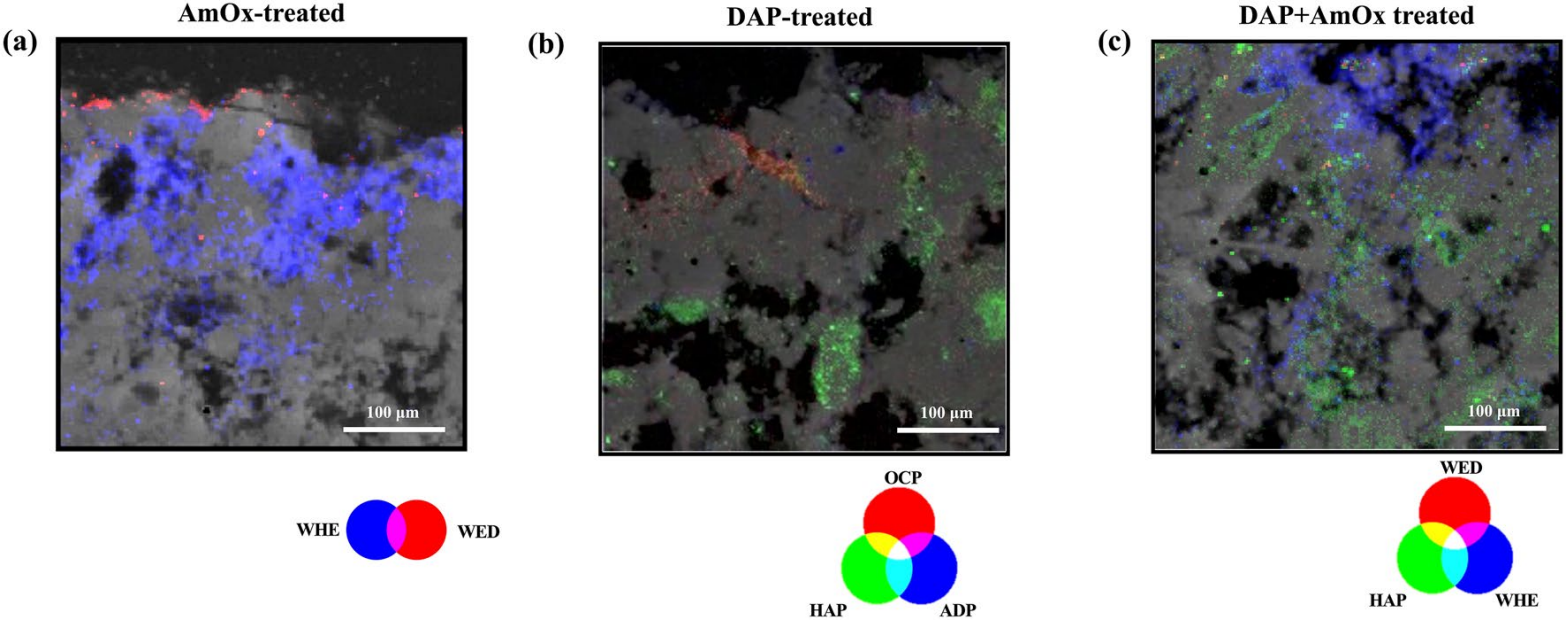
(**a**) RGB correlation of the μXRPD areal distribution maps of whewellite (WHE) and weddellite (WED) in an AmOx-treated sample; (**b**) RGB correlation of the μXRPD areal distribution maps of the marker peaks of identified HAP and by-products (ADP and OCP) in a DAP-treated sample; (**c**) RGB correlation of the μXRPD areal distribution maps the main CaOxs (whewellite and weddellite) and CaPs (hydroxyapatite) in a DAP + AmOx treated sample. All three figures are an ROI of 400 × 400 μm^2^ taken from the surface of the samples.

In the context of AmOx-treated samples, the areal distribution data through RGB correlation maps reveals a distinct distribution pattern of CaOx phases: a gradient from the surface, with weddellite (WED) overlaying whewellite (WHE). This observation underscores the spatial relationship between these phases shown in Fig. 6a. The data underscored a crystalline network following the solution diffusion path within the stone’s microstructure, originating from the surface interface. These findings highlight the CaOx areal arrangement given by the treatment. Such data offer, for the first time, an opportunity to establish correlations between the phases of CaOx formed and their spatial distribution, allowing interpretations of the consolidation effect. Currently, literature only suggests that the distribution and penetration of distinct CaOxs significantly affect resultant system properties^49^.

The application of μXRPD mapping of DAP reaction products has revealed unprecedented details regarding the areal distribution of CaP phases within the stone, particularly highlighting hydroxyapatite (HAP) and its associated by-products (ADP, OCP, and C-HAP). As shown in Fig. 6b, HAP distribution follows a gradient from the surface, less homogeneous than the CaOxs network. The by-products, less identified in prior studies, exhibit a distribution pattern linked to HAP, extending to similar depths within the stone and localized in spots within its porous network. It is important to acknowledge that the representation of these CaP phases in the color maps might be subject to potential inaccuracies (either underestimated or overestimated) because of the mixture of phases within our systems. CaP peaks are collocated in the diffraction pattern close to the peaks of other phases complicating their isolation and mapping. Despite this, the ability to pinpoint the spatial distribution of both HAP and its by-products remains crucial. The literature already suggests correlations between the presence of these by-products, such as ADP indicating acidification, and changes in the stone’s condition^10^. Therefore, having data on CaP distribution allows for a deeper exploration of these correlations, facilitating a better understanding of the localized effects of CaP phases on the stone’s chemical changes and conditions.

In the case of sequential treatment, the mapping of CaOxs and CaP phases, and the examination of phase coexistence through RGB correlation maps, highlight distinct variations in both penetration depth and areal distribution compared to single treatments. The data (Fig. 6c) showed that, in sequential treatments, HAP and by-products are dispersed throughout the stone bulk rather than follow a gradient from the stone surface. CaOxs seemly exhibit different penetration depths compared to single treatments, although they are still primarily located on the surface. Additionally, the maps illustrate sparse areas of coexistence between CaOxs and CaPs. These observations strongly indicate interactions between the solutions’ reactions. The significance of obtaining these data lies in its capacity to shed light on these interactions, thereby enhancing our understanding of combining treatment effects on stone microstructures absent in the literature.

### Quantitative phase analysis and orientation of the reaction products

The high resolution of μXRPD maps also enables us to obtain information on quantitative phase concentration and orientation at the scale of the studied area (ROI = 400 × 400 μm^2^). Acquiring such data at this scale, with a clear view of the phases’ distribution, provides us with essential insights for assessing localized effects and evaluating treatment performance.

For quantitative assessment of newly crystallized reaction products, image analysis of phase-specific μXRPD maps was used to quantify the areal fraction of reaction product observed in the 2D map (as explained in the Methods paragraph). The % ± Standard Deviation results are shown in Table 1. In the case of AmOx-treated samples, this quantification confirmed existing literature by highlighting predominant whewellite crystallization, while uncovering unexpectedly high content of weddellite in some surface areas. Similarly, in samples treated with DAP solution, through quantitative evaluation, we categorized the main reaction products (HAP and OCP, 22.2% and 7.6%, respectively) as well as reaction by-products into minor (between 1% and 0.1%) and trace (less than 0.1%) products, based on their concentrations. The localized quantitative data derived from μXRPD map image analysis, of the sequentially treated samples, revealed that the % of crystallization of the reaction compounds is, generally, in line with the one in the single treatments.

**Table 1:** Quantitative assessment based on image analysis of the reaction products areal fraction  (Mean % ± Standard deviation) on surface maps for various treatment methodologies.

	Reaction products					
	WHE	WED	HAP	C-HAP	OCP	ADP
AmOx-treated	25 ± 2.7	1.5 ± 0.5	–	–	–	–
DAP-treated	–	–	22 ± 3.9	1.5 ± 0.6	7.6 ± 1.4	0.3 ± 0.1
DAP + AmOx treated	21 ± 3.5	2.8 ± 0.6	16 ± 2.2	1.2 ± 0.4	5.4 ± 1.7	0.5 ± 0.2

**Figure 7:**
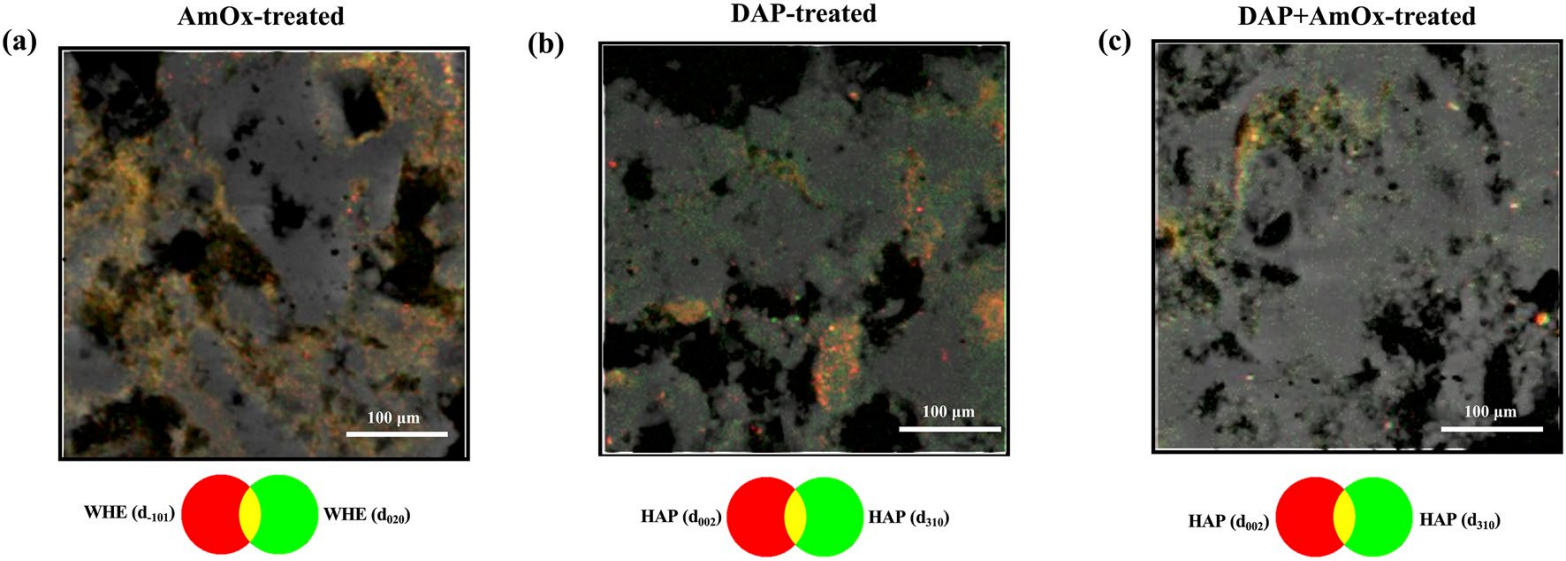
(**a**) RGB correlation of the μXRPD areal distribution maps of two marker peaks of whewellite in an AmOx-treated sample; (**b**) RGB correlation of the μXRPD areal distribution maps of two marker peaks of hydroxyapatite in a DAP-treated sample; (**c**) RGB correlation of the μXRPD areal distribution maps of two marker peaks of hydroxyapatite in a DAP + AmOx treated sample.

While generating μXRPD distribution maps by selecting Bragg peaks corresponding to the crystalline phase, we observed variations among maps for different diffraction peaks. Each peak in the diffraction pattern represents specific crystallographic planes, mapping the areal distribution of distinct crystal planes. RGB correlation of maps associated with different peaks of a single phase was employed to reveal the crystal orientation of the reaction products. The lack of color merging in specific areas in RGB correlation maps indicated the crystal orientation of selected crystalline planes. For instance, in the case of CaOxs (both whewellite and weddellite), they exhibit randomly distributed crystallites within the stone matrix, showing up as a single cohesive yellow color on the maps (Fig. 7a). On the other hand, CaPs, primarily HAP, in DAP-treated samples show preferential orientations in some areas, as distinct green (HAP d_310_) and red (HAP d_002_) color distributions show up on the correlation maps (Fig. 7b). This observation becomes particularly intriguing when studying sequential treatments where no significant orientation of CaPs can be detected (Fig. 7c). Here the data show how CaPs crystallized differently. This proves an interaction between the solutions (the DAP-stone system is disturbed by the crystallization of CaOxs,) and the reactivity of the CaPs phases to be disturbed by the interaction.

Overall, our experimental setup prioritized high spatial resolution and simultaneous μXRF and μXRPD acquisition, but this compromised the features of the collected X-ray diffraction patterns hindering, for example, a proper Rietveld analysis. The preclusion of Rietveld’s full-profile fit hinders any structural analysis of the crystalline phases, along with a phase quantification (in a multi-phase system) based on the refined scale factor of each crystalline component. However, this limitation is here efficiently overcome, as 1) the crystal structure of the co-present crystalline phases, in the system under investigation, is already well known and 2) the relative (areal) fraction of the crystalline components can be obtained through the μXRPD map analysis, along with their average crystallites orientation. In this light, the lack of the Rietveld full-profile fit does not diminish the description of the complex polycrystalline system under investigation and opens a new route for other scenarios in which the application of full-profile fit analysis is precluded.

## Conclusion

Our research effectively demonstrates how the synergy of μXRF and μXRPD at high resolution leads to an efficient characterization of the newly formed conservation products (CaOxs and CaPs), overcoming the analytical challenge of their low wt% concentration compared to the dominant calcite matrix. While μXRPD alone struggled, due to their low abundance, μXRF provided elemental data, leading to *area-specific* diffraction patterns, and ultimately enabling phase identification in complex multiphase mixtures.

Moreover, these techniques provide invaluable insights into the penetration depth and spatial distribution of reaction products, enhancing our understanding of the treated stone system. The data derived from μXRPD mapping proved, for the first time, to be crucial in assessing reaction product concentrations. Additionally, orientation analysis of crystalline phases on the μXRPD maps offered a deeper understanding of phase formation and preferential orientations within the treated samples, and of the phase interaction in the case of sequential treatment.

While acknowledging some limitations, mainly due to the need for micro-sampling, the combination of the techniques provided high-quality data on CH stone consolidation. Moreover, the obtained positive outcomes not only contribute to our fundamental understanding of these complex systems and inorganic mineral treatments but also underline their potential applications in further studies.

In conclusion, this study underscores the significance of combining advanced synchrotron-based X-rays 2D mapping techniques for comprehensive and spatially resolved analyses of complex CH multiphase systems, which broadens the applicability of these advanced synchrotron-based approaches in the study of inorganic mineral treatments in Conservation Science and Heritage Science.

## Data Availability

The data used and analyzed during the current study are available from the corresponding author upon reasonable request.
